# Uniform versus Asymmetric Shading Mediates Crown Recession in Conifers

**DOI:** 10.1371/journal.pone.0104187

**Published:** 2014-08-19

**Authors:** Amanda L. Schoonmaker, Victor J. Lieffers, Simon M. Landhäusser

**Affiliations:** 1 Boreal Research Institute, Northern Alberta Institute of Technology, Peace River, Alberta, Canada; 2 Department of Renewable Resources, University of Alberta, Edmonton, Alberta, Canada; University of Vigo, Spain

## Abstract

In this study we explore the impact of asymmetrical vs. uniform crown shading on the mortality and growth of upper and lower branches within tree crowns, for two conifer species: shade intolerant lodgepole pine (*Pinus contorta*) and shade tolerant white spruce (*Picea glauca*). We also explore xylem hydraulics, foliar nutrition, and carbohydrate status as drivers for growth and expansion of the lower and upper branches in various types of shading. This study was conducted over a two-year period across 10 regenerating forest sites dominated by lodgepole pine and white spruce, in the lower foothills of Alberta, Canada. Trees were assigned to one of four shading treatments: (1), complete uniform shading of the entire tree, (2) light asymmetric shading where the lower 1/4–1/3 of the tree crown was shaded, (3) heavy asymmetric shading as in (2) except with greater light reduction and (4) control in which no artificial shading occurred and most of the entire crown was exposed to full light. Asymmetrical shading of only the lower crown had a larger negative impact on the bud expansion and growth than did uniform shading, and the effect was stronger in pine relative to spruce. In addition, lower branches in pine also had lower carbon reserves, and reduced xylem-area specific conductivity compared to spruce. For both species, but particularly the pine, the needles of lower branches tended to store less C than upper branches in the asymmetric shade, which could suggest a movement of reserves away from the lower branches. The implications of these findings correspond with the inherent shade tolerance and self-pruning behavior of these conifers and supports a carbon based mechanism for branch mortality – mediated by an asymmetry in light exposure of the crown.

## Introduction

Light availability is an important driver of plant growth and crown development, particularly in multilayered forests [1]–[3]. Under shaded conditions, shade-intolerant species generally allocate carbon to height growth in order to evade shaded areas, potentially at the expense of allocation to other important tissues such as roots and leaves [4]–[6]. Shade-tolerant species distribute carbon more proportionally within the whole plant, but with a preference towards photosynthetic tissues to increase light capture under shaded conditions [4], [6].

In closed-canopy forests, light limitation is a significant driver of lower branch mortality and crown recession for trees [7], [8]. As it relates to carbon (C), it is also thought that branches in a crown behave as autonomous units [9], as there is little evidence for long-distance C movement between branches within a crown [10]–[17]. There is growing evidence, however, that C limitation due to reduced light is not the only driver of branch mortality, especially in large trees. Other factors such as nutrient limitation [18], [19]; hydrological constraints [20], [21], and heterogeneity in light within crowns [22]–[24] have also been linked to the mortality of lower branches, which makes branch recession a more complex issue than previously thought.

Shedding lower branches may be advantageous for the survival of the whole plant under conditions of competition (for example during stem exclusion in forest development). Particularly with shade-intolerant species, the drive to reach high light may overwhelm all other growth responses. This was observed in *Pinus taeda* [5] and in forest stands of *Pinus contorta* where biomass allocation was focused in the upper crown [25]. In addition, for species that exhibit crown shyness (eg. *Pinus contorta* [26]), maintenance of lower branches would constitute a poor use of resources.

Sprugel [23] showed that a suppressed (fully shaded) individual of the shade-tolerant amabilis fir (*Abies amabilis* (Dougl.) Forbes) carried live branches in a light environment that would normally result in the mortality of lower branches for trees in a dominant canopy position where the lower portion of the crown is shaded. Similarly, in a *Betula pubescens* ssp. *czerepanovii* (N.I. Orlova) seedling, shading one of two branches resulted in reduced growth and increased mortality compared with complete shading of both branches [22]. These examples both suggest that the relative differences in light levels between crown positions may play as important a role in driving branch mortality (or crown recession) as the absolute quantity of light. If the strength of a resource sink is a function of growth activity and proximity to a source [9], then partial shading may actually create greater disparity between sink strength in upper (illuminated) branches and lower (shaded) branches. Quantification of non-structural carbohydrates may provide further insight to relative differences in source or sink strength between branch positions.

Given the inherent differences in growth strategies under low-light, it is plausible that light-mediated crown recession might also operate differently between species of opposing shade tolerance. In this study we systematically explore the impact of asymmetrical vs. uniform crown shading on the mortality and growth of upper and lower branches within tree crowns, for two conifer species of differing shade tolerance. We hypothesize that the shading of only the lower crown, as compared to the entire crown, will have a larger negative impact on the lower branches in the shade intolerant lodgepole pine (*Pinus contorta* Douglas ex. Louden) than in shade tolerant white spruce (*Picea glauca* (Moench) Voss). This hypothesis was experimentally tested in a large-scale field trial with juvenile trees (∼17 years old). Given that shading or branch position has been shown to impact a variety of physiological parameters within trees including nutrition [18], [19] and hydrological constraints [20], [21]; we also explore xylem hydraulics, foliar nutrition and carbohydrate status as drivers for growth and expansion of the lower and upper branches under partial and full crown shading.

## Methods

### Study sites

Ten study sites were selected in the lower foothills natural subregion [27] approximately 40–70 km north of Whitecourt, Alberta, Canada (54.14°N–115.68°W). Site elevations ranged from 840–960 m. Average annual precipitation in this region is 578 mm and annual precipitation during the study period was 439 mm in 2008 and 438 mm in 2009. Daily average temperature is 2.6°C with a mean monthly temperature of −12.1°C in January and 15.7°C in July [28]. The selected sites were areas that had been harvested in 1991–1992 and were planted with or naturally regenerated to lodgepole pine, white and black spruce (*Picea mariana* (Mill.) Britton, Sterns & Poggenb.). Sites were located over a 100 km^2^ area. Early silvicultural treatments included herbicide applications to reduce competition from hardwoods and some density management through pre-commercial thinning. Densities of conifers in 2008 ranged from 500–2250 stems ha^−1^. Within each site, 3–4 white spruce and lodgepole pine each were randomly selected from a pool of twenty trees identified as being ‘well-spaced’ in that neighboring trees were more than 2 m from the target trees. Permission to study these trees was provided by the local forest management agreement holder (Blue Ridge Lumber, Blue Ridge Alberta). Shade treatments were applied in spring of 2008 (described below). The average height of selected pine in spring 2008 was 3.4 m (0.4 SD) and spruce was 3.3 m (0.4 SD). At the termination of the experiment (October 2009), the average height of the pine was 4.5 m (0.4 SD) and spruce was 4.4 m (0.4 SD).

### Experimental design

Trees were assigned to one of four shading treatments: (1), complete uniform shading of the entire tree (US) with a single layer of saddle tan shade cloth (Easy Gardener Products Inc., Waco Texas USA), (2) light asymmetric shading (AS-L) where the lower 1/4–1/3 of the tree crown was shaded with a single layer of shade cloth, (3) heavy asymmetric shading (AS-H) as in (2) except the lower crown was shaded with a second layer of shade cloth (black fiberglass insect screen) and (4) control (NS) in which no artificial shading occurred and most of the entire crown was exposed to full light. Treatment 3 was applied to trees at only 6 sites and the other treatments were applied at all 10 sites. Asymmetric shading (treatments 2 and 3) involved the construction of a self-supporting wooden structure around the lower branches of each tree, covered with shade cloth (Appendix S1, The individual in this manuscript has given written informed consent (as outlined in PLOS consent form) to publish these case details). Shading the entire tree was accomplished by building a cone-shaped wooden structure (teepee) that was covered with shade cloth (Appendix S1). All structures were large enough to minimize the abrasion of branches and accommodate any future growth during the two years of study. According to the manufacturer, the saddle tan shade cloth reduces ultraviolet rays by 81–87% and reduced incoming light by 70–75%. The shade cloth was permeable to rain, however, due to self-shading and surrounding neighbor trees the actual light reduction was greater than 85% (Table 1). It was previously demonstrated that air temperature within the crowns of shaded (using black fiberglass insect screen) Pinus contorta was within 1.1°C of unshaded crowns [29]. Each year shade cloth was installed at the onset of shoot and needle expansion and removed in late-September to avoid damage due to snow-loading. The shade treatments were applied in late May in 2008 and early May in 2009. To allow for acclimation of the trees to the different shade treatments, measurements of growth and physiology were taken during the 2009 growing season.

**Table 1:** Summary of average light level (expressed as a percentage of full light) in upper and lower crown positions.

	NS	US	AS-L	AS-H
Percentage of full light				
upper crown	82.7 (68.1–100.0)	14.3 (13.5–15.2)	-	-
lower crown	57.9 (42.8–71.9)	10.8 (7.6–12.8)	7.1 (2.3–9.5)	2.9 (0.8–5.6)
Mean percentage of light saturated photosynthesis				
*Pinus contorta*				
upper crown	100.0	41.2	-	-
lower crown	94.1	29.4	20.6	1.2
*Picea glauca*				
upper crown	100.0	45.0	-	-
lower crown	95.0	30.0	22.0	5.0

Measurements were conducted between 11:30–16:00 hours in mid-summer. Mean percentage of light saturated photosynthesis was estimated from light response curves ([Landhäusser and Lieffers 2001]) and PAR estimates determined from light reduction imposed by shading treatments. Treatment codes are as follows: NS = non-shaded, AS-L = asymmetric-light shaded, AS-H = asymmetric-heavy shaded and US = uniform-shaded. Values in brackets represent the range of measurements observed. Light was measured with multiple readings around the crown position with an Acupar Ceptometer.

### Sample collection and growth measurements

In 2009, lateral branches from both upper and lower positions were collected from the trees in June, late-August and October. These collections were made from the two most recent age classes of a branch: current-year (expanded in 2009) and one-year-old (expanded in 2008). In June, we collected a one-year-old internode from each tree for needle carbohydrate and nitrogen reserves; this time was chosen because it is a time of peak shoot growth and thus a period of high C and nitrogen (N) demand. In late August, the one-year-old section of a terminal shoot of the upper and lower branches of each species was collected in 6–8 replicate trees on the south aspect to determine hydraulic conductivity (k_h_) (see below). In October the two most recent age classes of four branches were collected (one from each cardinal direction) in both the upper and lower crown positions. The length of terminal shoots (current-year and previous year) were measured. To determine the frequency of bud expansion, we also counted the number of terminal buds for each branch that did or did not flush in the various treatments. In October during dormancy and just prior to the onset of ground frost, we also collected root samples (1 cm diameter) for root carbohydrate reserve analyses. These roots were collected within 10–15 cm of the soil surface by manually tracing roots from the stem base outwards until the appropriate diameter was found; this late collection was done to minimize disturbance to the tree during time of shade treatment.

### Carbohydrate and Nitrogen analyses

To analyze tissue samples (needles and roots) for total non-structural carbohydrates (water soluble sugars and starch), samples were immediately frozen on dry ice in the field, transported to the laboratory and stored at −20°C until further processing. Shoots were oven dried at 100°C for 1 hour and then 70°C until weight constancy. Twigs were separated from needles and the needles were ground to pass 40-mesh (0.4 mm) in a Wiley-Mill (Thomas Scientific, Swedesboro New Jersey, USA). Needles were used to represent TNC concentrations of the entire shoot as we had previously found that twig and needle TNC follow the same seasonal pattern though needles concentration was consistently higher (Schoonmaker et al., unpublished). Non-structural carbohydrates in tissues were quantified by boiling 50 mg of dried and ground tissue with ethanol (3 times) to extract the water soluble sugars followed by treatment with phenolsulfuric acid which breaks down sugars into monosaccharides, which are subsequently quantified colorimetrically. The remaining residue from the initial extraction was separately digested with enzymes (α-amylase and amyloglucosidase) in order to break down starch into glucose, which is then quantified colorimetrically [30]. Total nitrogen was determined using the Dumas combustion method [31] with a 4010 CHNS analyzer (Costech Analytical Technologies, Inc., Valencia, California). Soluble sugars, starch and nitrogen were presented as a percentage of total dry weight.

### Hydraulic conductivity

For the hydraulic conductivity (k_h_) of one-year-old terminal shoots, we followed the methodology described in Schoonmaker et al. [32], except that in the current study, the segments were 5 to 10 cm in length. Samples were refrigerated and measurements were conducted within four days of collection. Briefly, sealed hoses were connected to both ends of the shoot segments and a small pressure head of filtered (0.2 µm) 20 mM KCl+1 mM CaCl_2_ solution was applied; the outflow hose emptied into a sealed container on a balance (CP225D, Sartorius, Göttingen, Germany). After the rate of outflow stabilized, within 2–3 minutes, the average outflow over the next 40 s were used to calculate k_h_:
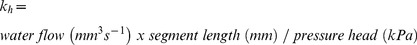
(1)Hydraulic conductivity was scaled to sapwood cross-sectional area to give sapwood-area specific conductivity (k_s_):
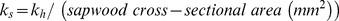
(2)Sapwood area was determined with a stereomicroscope (MSF, Leica, Wetzlar, Germany) and image analysis software (ImagePro Plus 6.1, Media Cybernetics, Silver Spring, MD, USA). As the plant tissues were young (2 years old), all wood tissue (except the pith) was assumed to represent conducting sapwood.

### Data analysis

All statistical analyses were carried out using R statistical software [33]. Linear mixed-effects models using shading treatment as the fixed effect and site as a random effect were used as a starting point for parameter estimates of treatment means and variances. Individual models were run for measurements conducted in upper and lower crown position as we were not interested in direct comparisons between crown positions but in relative changes within crown position due to shading treatments. Bootstrap simulations from these models were generated to obtain confidence intervals around treatment means and on the difference in means of NS from treated trees (AS-L, AS-H, US). Where subsamples within tree-crown position were collected (in growth October); individual branches were first averaged for each individual tree. Model assumptions were checked with diagnostic plots and where strong evidence of non-normality or unequal variance were observed, data were log-transformed (data presentation however, is based on back-transformed values).

Visual comparisons of the differences of means and their 95% confidence intervals were our main method of interpretation [34]–[36]. As a guide, we have included a comparison graph indicating the zero-line on the difference of means from the control. When confidence intervals of the mean difference intersect this line, it approximately corresponds to a p-value of >0.05 [37]. However, we have not limited our discussion of results to this somewhat arbitrary cut-off. As 95% confidence intervals of treatment means and associated differences between means provides a measure of the effect size of treatments [38], we believe this information will allow the reader to make their own judgment as to the statistical or biological relevance of the data.

## Results

### Expansion and growth

**Figure 1:**
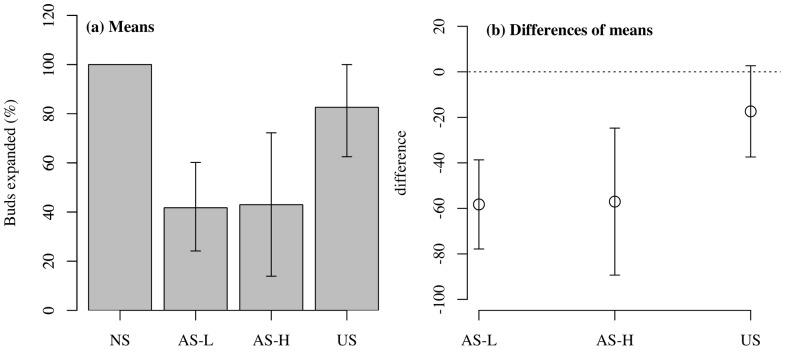
Branch survival of pine. (a) Frequency of bud expansion of current-year growth in the lower crown of pine collected after the second year of treatment (October 2009). Where NS = non-shaded, AS-L = asymmetric-light shaded, AS-H = asymmetric-heavy shaded and US = uniform-shaded. (b) Mean difference of shading treatments relative to un-shaded control (NS). Error bars represent 95% CI (n = 6–10).

In pine, the lower branches had a low frequency of bud expansion (∼40%) in both the AS-L and AS-H treatments after two growing seasons of shading, while in US trees, expansion was much higher (∼80%) and 100% in the NS trees (Figure 1). In contrast, frequency of bud expansion of the lower branches in spruce and upper branches of both species was 100% across all treatments (data not shown).

**Figure 2:**
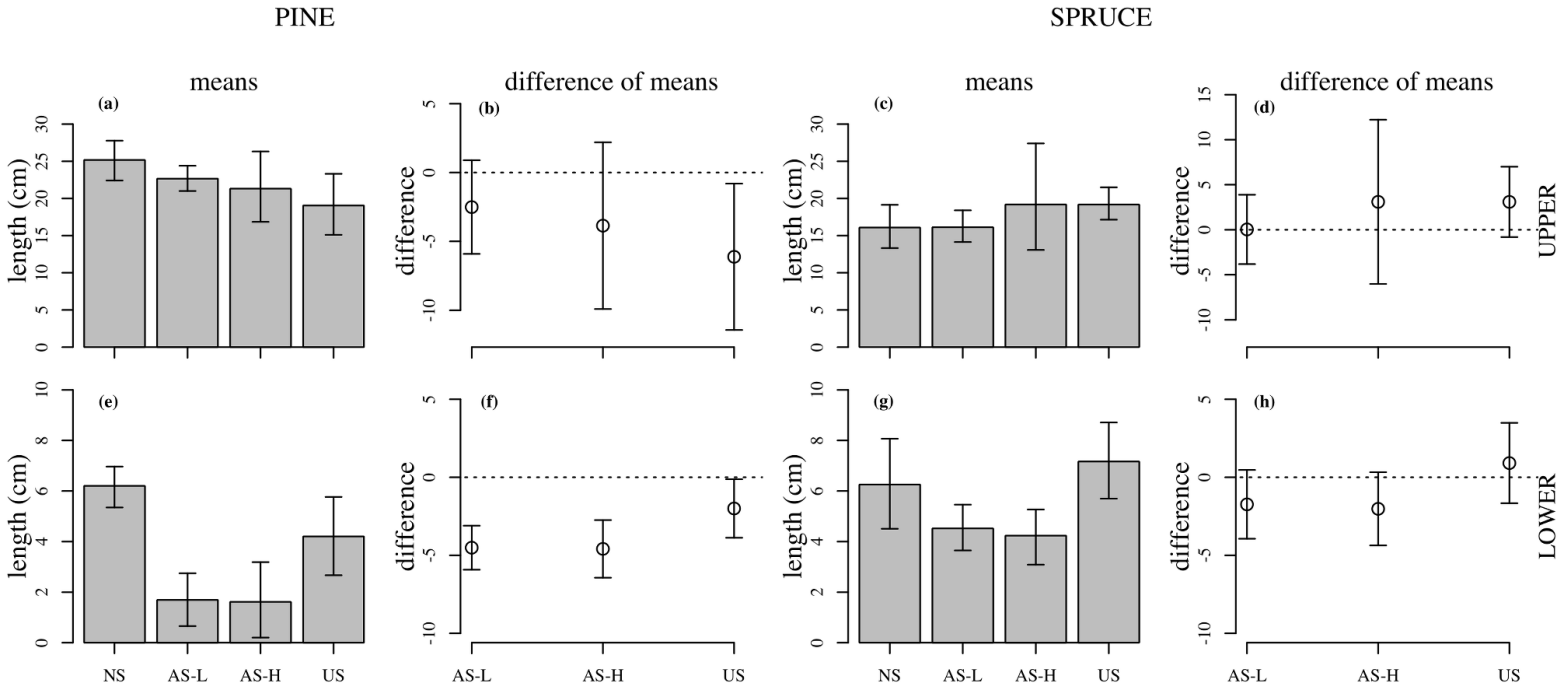
Shoot growth of pine and spruce. Growth in length of current-year shoots of pine and spruce from branches located in the upper (upper rows) and lower crown (lower rows) in October 2009. Note that length measurements were based on shoots collected on upper and lower branches in October (up to 4 shoots per treatment). The difference of means indicates the control minus the shade treatment. Treatment codes are represented as: NS = non-shaded, AS-L = asymmetric-light shaded, AS-H = asymmetric-heavy shaded and US = uniform-shaded. Error bars represent 95% CI (n = 6–10).

Terminal shoot growth of the upper branches of US pine trees was reduced by 6 cm compared with NS trees while asymmetrical shading (AS-H and AS-L) had little effect on the terminal shoot growth of the upper branches (Figure 2a,b). The growth of terminal shoots of the lower branches was 2.5 cm less in the US treatment compared to the control (NS) trees (Figure 2e,f); however, the terminal shoots of lower branches in both the AS-L and AS-H was 5 cm less compared to the NS control (>50% reduction in shoot growth) (Figure 2e,f). In spruce, terminal shoots of the upper branches of US trees grew 4 cm longer compared to shoots in the NS trees (Figure 2c,d). Lower branches in spruce showed no difference in shoot growth between the US and NS trees while asymmetric shading (AS-H and AS-L) of lower branches led to a 2.0 cm reduction in shoot length relative to lower branches of NS trees (Figure 2g,h).

### Carbohydrate reserves and nitrogen concentration

**Figure 3:**
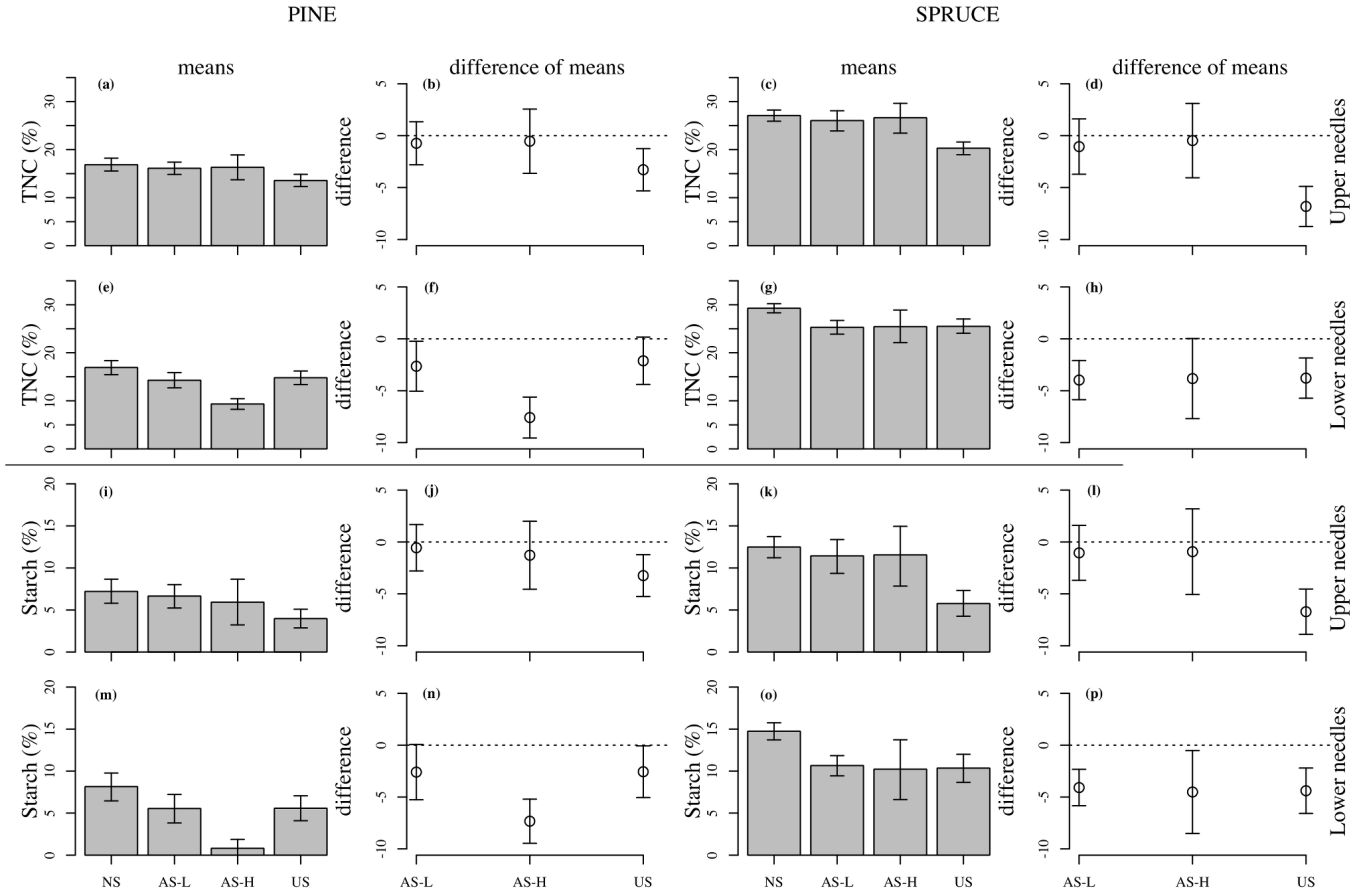
Total non-structural carbohydrates in pine and spruce needles. Total non-structural carbohydrates (TNC) and starch in previous year needles (one-year-old) of pine and spruce from branches located in the upper and lower crown in late June 2009. The difference in means indicates the control minus the shade treatment. Treatment codes are: NS = non-shaded, AS-L = asymmetric-light shaded, AS-H = asymmetric-heavy shaded and US = uniform-shaded. Error bars represent 95% CI (n = 6–10).

Total concentrations of nonstructural carbohydrates (TNC) in one-year-old needles (grown in 2008) of the upper branches of both species were only lower (relative to the control) in the US trees (Figure 3a–d). TNC concentrations in needles of the lower branches of pine were reduced by all shading treatments, especially in the AS-H treatment, where needle concentration were only 9% compared to 17% in the NS treatment (Figure 3e,f). In the spruce, needle TNC concentrations were uniformly reduced regardless of the shading treatment (Figure 3g,h). Starch concentrations in the one-year-old needles mirrored the changes in TNC concentrations in both upper and lower branches of both species (Figure 3).

**Figure 4:**
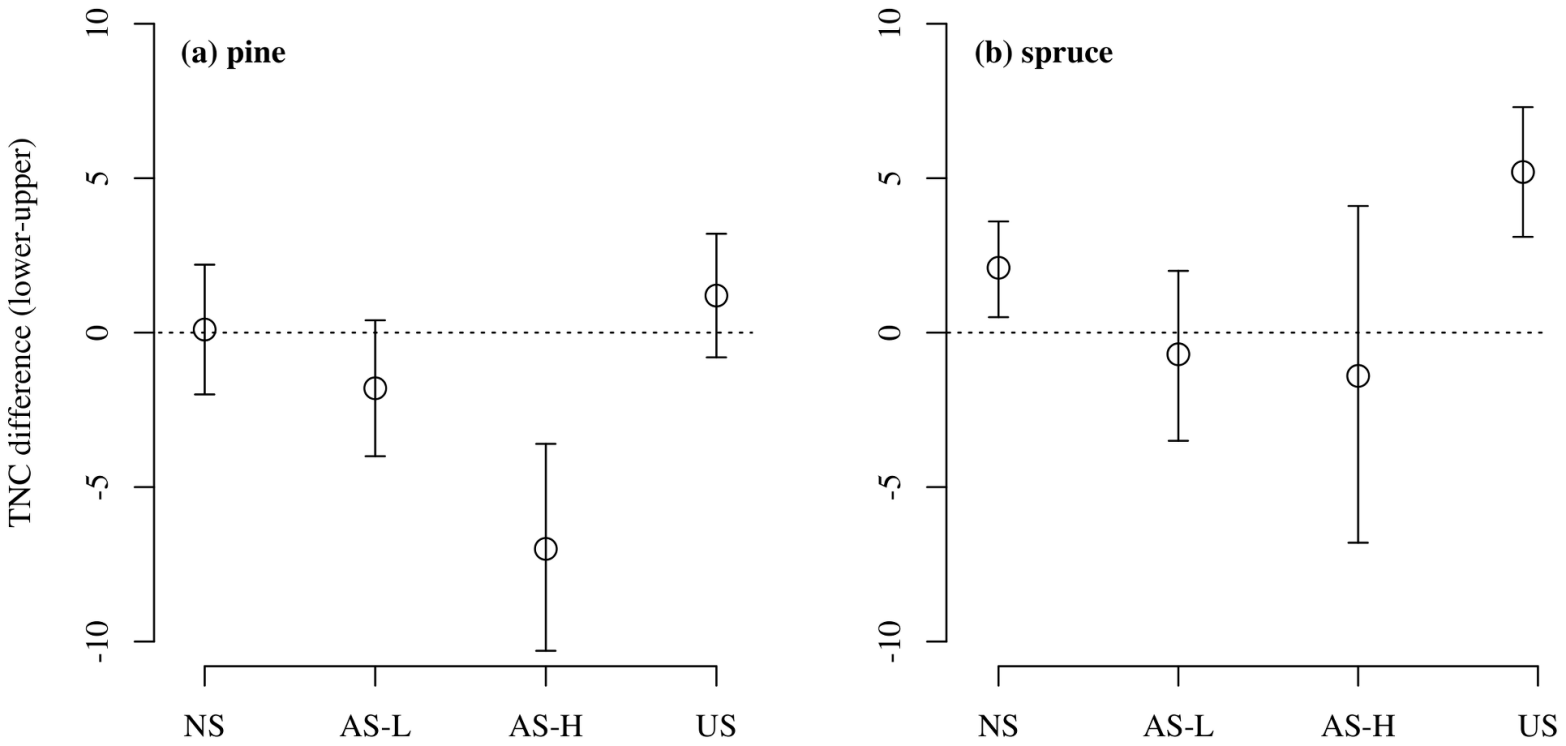
Difference in total non-structural carbohydrates in pine and spruce needles. Difference in total non-structural carbohydrate (TNC) of one-year-old needles between the lower and upper crown (lower- upper). Values below 0 indicate less TNC in lower than upper foliage. Treatment codes are: NS = non-shaded, AS-L = asymmetric-light shaded, AS-H = asymmetric-heavy shaded and US = uniform-shaded. Error bars represent 95% CI (n = 6–10).

To allow for comparison in the allocation of TNC relative to the NS trees, the differences in needle TNC concentrations between lower and upper branches (lower-upper) for one-year-old needles are presented in Figure 4. In pine, the needles from the lower branches had lower TNC concentrations compared with the upper needles in both asymmetric shading treatments (Figure 4a). In the US treatment needles on lower branches tended to have higher TNC concentrations relative to needles on upper branches (Figure 4a). In spruce, TNC concentrations of the needles on lower branches were higher than in the needles of upper branches in the NS and US treatment, but tended to be similar to the AS treatment (Figure 4b).

In pine, root TNC concentration was about 10% in the NS and AS treatments, but was only 8% in the US treatment (Appendix S2). For spruce, shading did not reduce root TNC concentrations (Appendix S2).

Nitrogen concentrations in the needles of lower branches of pine and spruce were similar across shading treatments (Appendix S3). Needles of the upper branches of pine were similar across shading treatments (Appendix S3) and in spruce both the AS-H and US trees had higher needle N than the control (Appendix S3).

### Hydraulic conductivity

**Figure 5:**
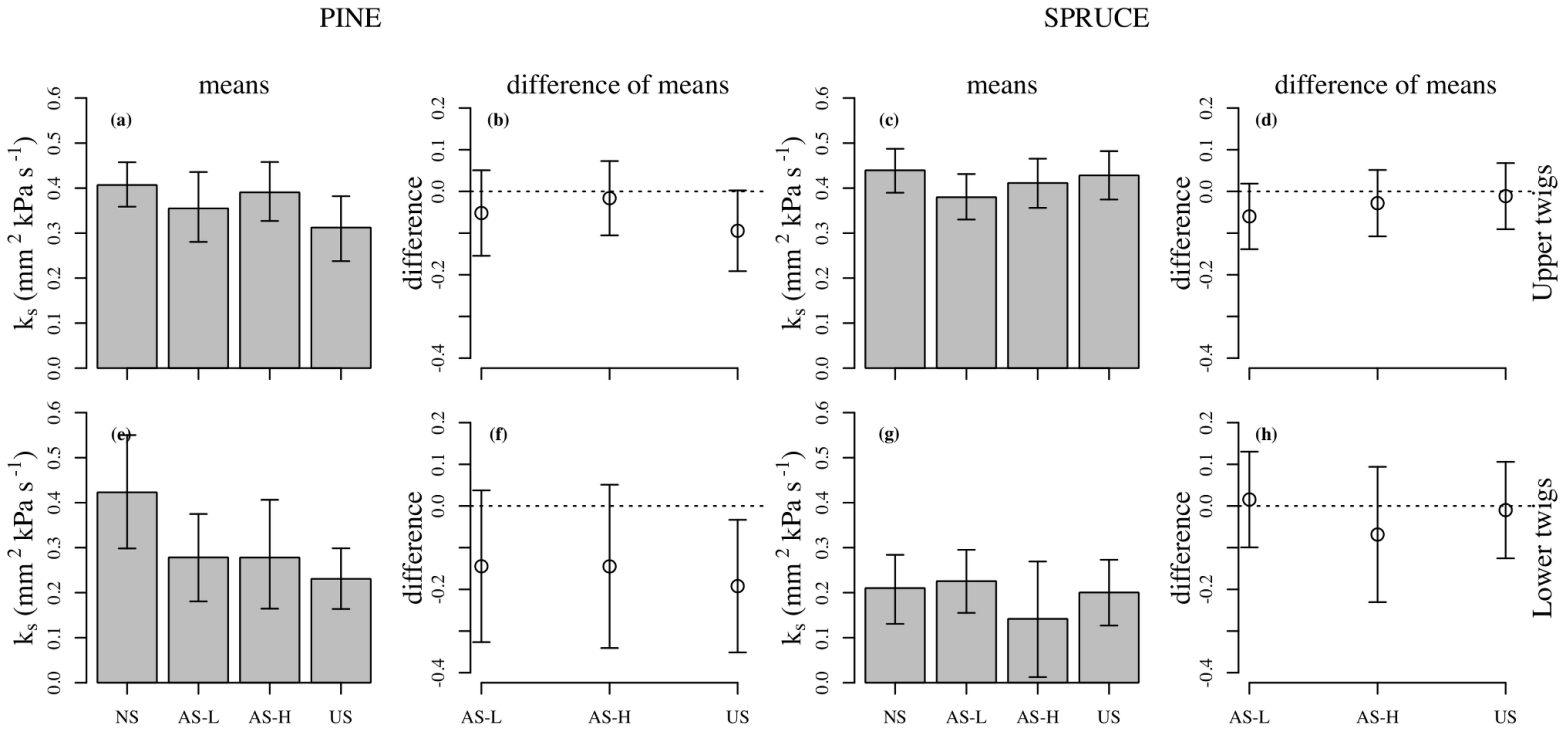
Sapwood area specific conductivity in one-year-old shoots of pine and spruce. Sapwood area specific conductivity in one-year-old shoots of pine and spruce collected from upper (upper row) and lower branches (lower row) of the crown in October 2009. The difference of means indicate the control – the shaded treatment. Treatment codes are represented as: NS = non-shaded, AS-L = asymmetric-light shaded, AS-H = asymmetric-heavy shaded and US = uniform-shaded. Error bars represent 95% CI (n = 6–10).

Shoots of the upper branches in pine had lower sapwood area specific conductivity (k_s_) in the US treatment compared to the other treatments (Figure 5a,b). Shading had little impact on k_s_ in the upper or lower branches of spruce (Figure 5c,d,g,h). In the lower branches of pine, k_s_ declined up to 40% in all shading treatments (Figure 5e,f).

## Discussion

**Figure 6:**
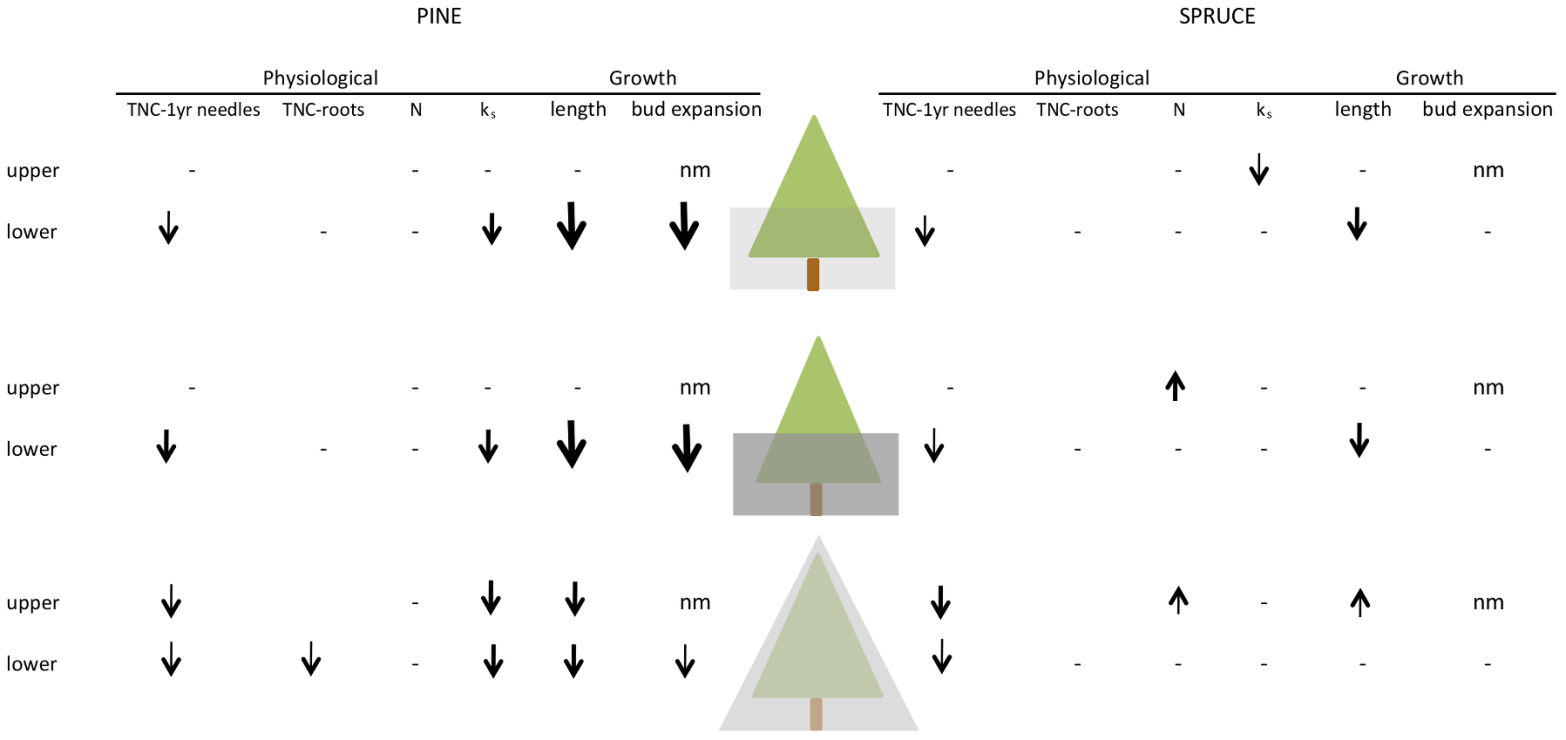
Summary of effects of shading treatments in pine and spruce. Summary of effects of shading treatments on growth, expansion and physiological parameters, in pine (*Pinus contorta*) and spruce (*Picea glauca*). The top row (tree) corresponds with asymmetrical-light shading, the middle row (tree) with asymmetrical-heavy shading and the lower row (tree) with uniform shading. ‘nm’ indicates that no measurement was not taken, a dash (-) indicates no difference between non-shaded control tree and shaded tree, a downward arrow (**
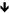
**) indicates a reduction and an upward arrow (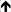) an increase as a result of shading. Small non-bolded arrows indicate <20% changes, small bolded arrows indicate 20–50% change and large bolded arrows >50% change relative to non-shaded trees.

Overall, our study suggests that crown recession (lower branch mortality) is not only driven by the quantity of light, but also by the relative difference in light between the lower and upper branches (see also Figure 6). Asymmetrical shading of the lower crown (AS) had a larger negative impact on bud expansion and growth than did uniform shading of the whole crown (US). This effect was strongest in the shade intolerant lodgepole pine. These strong reductions in growth and bud expansion observed in the lower AS branches of pine are consistent with a more severe self-pruning behavior observed in closed-canopy pine stands through reduced live crown ratios [4]. The impact of AS vs. US on the lower branches of the shade-intolerant pine (and to a lesser extent in spruce) is also consistent with observations made in potted seedlings of *Betula pubescens*, a shade-intolerant deciduous species [22]. Similar ideas have been generated in more indirect studies on *Cedrela sinensis* A. Juss, a deciduous pioneer species [24], *Litsea acuminata* (Bl.) Kurata, an evergreen broad-leaved understory tree [39] and other conifers [23]. The strong reaction to asymmetric shade in lower branches of the intolerant pine may be indicative of the fundamental differences in shade tolerance among species. Our contribution to this topic relates to the understanding of the mechanisms on why disparity in light among branches within a crown is detrimental to the health and longevity of the shaded branches.

The average percentage of full light available in the lower branches of US and AS-L were intended to be the equivalent although mean values for US were 10.8% and 7.1% for AS-L. There was however, overlap in the range of values (Table 1) and the photosynthetic responses at these levels suggests a much larger effect between AS-H and AS-L where the percentage of light saturated photosynthesis in AS-H pine is 1.2% and 5.0% in spruce (Table 1). Moreover, we did not observe any differences in TNC between US and AS-L, further supporting that the light levels experienced between these treatments were similar enough but that the AS-H light reduction was more severe (Figure 4). Changes in light quality due to the shade material utilized may also influence photosynthetic and growth responses of developing shoots. It is well-known that red∶far red ratio (R∶FR) does decline in closed canopy forests [40] with declining light transmission. As only the PAR region was quantified, we cannot comment directly on any light quality shifts in the R∶FR region due to the shade cloth though it is likely less than that observed in forest stands as the canopy preferentially absorbs red light for photosynthesis. Moreover, Kitajima [41] has found that even with changes in R∶FR ratio, light intensity played a more significant role in determining photosynthetic and growth related changes in a range of tropical species.

In both species, the relative difference in TNC reserves between the needles of the upper and lower crown may provide an explanatory mechanism for the observed distinction between the growth responses of the lower branches of AS and US (and reduced occurrence of bud expansion in pine). In pine, the difference in TNC between the needles of lower branches and upper branches was greatest in the AS-H, followed by the AS-L (Figure 4); which suggests a greater TNC difference between the shaded zone and the well-lit upper zone. In the NS and US trees there was no difference in TNC concentration between needles of the upper and lower shoots. Particularly for pine, this suggests that under AS, TNC could have been (1) moved out of the newer needles of lower shaded branches to other growth or maintenance sinks and/or (2) there was simply less TNC being accumulated in lower branches due to reduced photosynthesis (as suggested by Sprugel [23]). As carbon movement between branches is generally more limited [10]–[17], however note the comment below regarding bud flush, it is more likely that the second mechanism was the primary cause for the pine in this study. In spruce, TNC was higher in lower branches of NS and US branches relative to upper branches with no difference in TNC in AS shading treatments between branch positions. Higher TNC in lower branches may encourage stronger sink-strength to these branches [9], [42]; resulting in lower branches being in a favorable competitive position with well-illuminated upper branches for other resources such as water, nutrients or potentially carbon during periods of tree-wide carbon movement such as bud flush [9], [43], [44]. This also appears to be a more conservative system for the maintenance of lower branches (and leaf area) in a shade tolerant species.

Spruce maintained intrinsically much higher needle carbohydrate levels (>30% more) than pine, providing this species with a larger reserve storage buffer that likely allowed it to extend growth in the shade. In pine, even the lower branches of NS trees maintained average TNC values of only 17% (compared with nearly 30% in spruce) during the growing season, and in both AS pine treatments, TNC concentrations in needles of the lower branches were between 10–15%. The reliance of shaded trees on stored TNC reserves was also shown in seedlings of tropical species, where seedlings with the highest TNC concentrations had the greatest shoot expansion when exposed to deep shade [45].

The reduced levels of bud flush and shoot development in the lower branches of pine (particularly in the AS treatments) might also be related to the lower needle carbohydrates, as shoot flush and shoot expansion has been linked to C supply from nearby needles [10]. Poor development of shoots was particularly evident in the lower branches, where one-year-old shoots of AS-H pine showed an almost a 50% decline in TNC. Though both lower than NS trees, TNC in lower one-year-old shoots of AS-L and US trees were similar in concentration. However, they were vastly different in terms of expansion and growth (US shoots on lower branches performing better). Therefore, C available in the AS-L branches at this time of year is not completely indicative of bud expansion and the eventual fate of the distal shoot.

Carbohydrate reserves in the pine roots were reduced in the US treatment (particularly so for starch), but in spruce, root TNC reserves were not affected by the shade treatments over the two growing seasons. In fact, there was more starch stored in roots of the AS treatments – although retention of the root starch late into the fall may have been related to warmer soils under the shade cloth. The long-term consequence of reducing reserves to roots in the US pine could result in a negative feedback loop on root growth and expansion including reduced uptake of water and nutrient [46]. Souza and Válio [6] similarly observed a decline in the translocation of radioactively labeled carbon to roots in forest-shaded (versus fully-illuminated) early-successional seedlings of the tropical species *Cecropia pachystachya* Ambay and *Schizolobium parahyba* (Vell.) S.F.Blake. This suggests that reduced C allocation to roots in light-deprived trees may be an important component of shade intolerance. In the more shade tolerant spruce, root TNC and starch concentrations were maintained despite the overall lower C status of the trees in the US treatment.

Sapwood area specific conductivity (k_s_) declined in the shaded lower branches and upper branches (US) of pine, while shading treatments caused little change the k_s_ of lower branches in the spruce. It is likely that the reduction in conductivity under low light would correspondingly reduce photosynthesis [47]. Low light conditions may also increase the susceptibility of trees to drought through reductions in cavitation resistance [48], [32], [49]. Low light has been attributed to reduced conductivity in previous studies of *Pinus contorta* [21], *Pseudotsuga menziesii* (Mayr) Franco and *Tsuga heterophylla* (Raf.) Sarg. [46]. [Kupper et al. [2006]] also observed declines in branch conductance and transpiration of fully-illuminated lower branches compared with upper branches of *Larix decidua* Mill. Both the current study, as well as those described above, suggests an additive effect of branch position and light on water relations. Reduced conductivity may be detrimental for lower branches, however, it is also clear that transpirational demand will concurrently decline in low light conditions and therefore there may be less ‘need’ to produce xylem with high transport capacity. It is, however, unclear if the production of xylem with reduced water capability is an adaptive response to reduced water stress or simply a consequence of low-light and lowered capability to produce xylem. In the current study, a water-relations related mechanism appears to only be apparent for the shade intolerant pine.

Surprisingly, we observed no change in N concentration in the lower branches of both species even though needle N concentrations indicated N was deficient on these sites [51]. This suggests that with these conifers, there was little extraction of N out of lower branches under shaded conditions. Yoshimura [24] similarly did not observe reduced N in leaves of partially or fully shaded *Cedrela sinensis* saplings. Nonetheless, periods of N limitation have been associated with N translocation from the lower to upper crown in *Eucalyptus* [19]. Livingston et al. [52] also observed increased N concentration with height in *Pinus radiata* D.Don that was not attributable to changes in light. Strangely, we did observe an increase in N content in upper branches in AS-H and US treatments of spruce (but not in AS-L); given that we did not observe a concurrent reduction in N of lower branches, this was a puzzling result. Though not significant, both of these treatments also exhibited slightly higher increases in shoot growth, this could conceivably have triggered greater N demand and increased overall N content.

In summary, our study showed two strong physiological differences between pine and spruce which may indirectly affect lower branch survival in shade. The first difference being lower inherent levels of shoot and root carbohydrate reserves in pine relative to spruce, which may make pine less resilient to stress. Secondly, a decline in sapwood-area specific conductivity of lower branches in pine where any type of shading would limit the rate of water movement within shoots. We saw no difference in foliar N in response to shading in lower branches in either species. The only clear evidence across both species that asymmetric shade is more stressful to lower branches than uniform shade relates to carbohydrate storage. In both species, the needles of lower branches tended to store less C than upper branches under asymmetric shade and this effect was strongest in pine. Thus resources were more limiting in lower branches under asymmetric than under uniform shade, thereby make them more vulnerable to mortality.

## Supporting Information

Appendix S1 Photographs of the (a) asymmetric shading treatment structure (before the shade cloth was applied) in *Picea glauca* and (b) uniform shading treatment on *Pinus contorta*.
(TIF)

Appendix S2 Total non-structural carbohydrate concentration (TNC, upper row) and starch (lower row) in roots of pine and spruce in October 2009. The difference of means indicates the control minus the shaded treatment. Treatment codes are represented as: NS = non-shaded, AS-L = asymmetric-light shaded, AS-H = asymmetric-heavy shaded and US = uniform-shaded. Error bars represent 95% CI (n = 6–10).
(TIF)

Appendix S3 Nitrogen concentration in one-year-old needles and shoots of pine and spruce collected from branches in the upper (upper row) and lower (lower row) crown in late June 2009. The difference of means indicate the control – the shaded treatment. Treatment codes are represented as: NS = non-shaded, AS-L = asymmetric-light shaded, AS-H = asymmetric-heavy shaded and US = uniform-shaded. Error bars represent 95% CI (n = 6–10).
(TIF)
