# Supplementary figures and images for: Uniform versus Asymmetric Shading Mediates Crown Recession in Conifers
Source: PLoS One. 2014 Aug 19;9(8):e104187. doi: 10.1371/journal.pone.0104187 (PMC4138101; doi:10.1371/journal.pone.0104187)

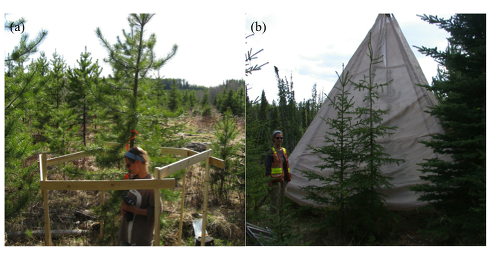

Supplement: Appendix S1 — Photographs of the (a) asymmetric shading treatment structure (before the shade cloth was applied) in Picea glauca and (b) uniform shading treatment on Pinus contorta. (TIF) [file pone.0104187.s001.tif]
